# Dietary Patterns and Cognitive Function among Older Community-Dwelling Adults

**DOI:** 10.3390/nu10081088

**Published:** 2018-08-14

**Authors:** Erin L. Richard, Gail A. Laughlin, Donna Kritz-Silverstein, Emilie T. Reas, Elizabeth Barrett-Connor, Linda K. McEvoy

**Affiliations:** 1 Department of Family Medicine and Public Health, University of California San Diego, 9500 Gilman Drive, La Jolla, CA 92093, USA; glaughlin@ucsd.edu (G.A.L.); dsilverstein@ucsd.edu (D.K.-S.); ebarrettconnor@ucsd.edu (E.B.-C.); lkmcevoy@ucsd.edu (L.K.M.); 2 Department of Radiology, University of California San Diego, 9500 Gilman Dr, La Jolla, CA 92093, USA; emiliereas@gmail.com

**Keywords:** alternate healthy eating index, cognitive function, dietary patterns, exploratory factor analysis, Mediterranean diet, Rancho Bernardo Study

## Abstract

Diet may be an important modifiable risk factor for maintenance of cognitive health in later life. This study aimed at examining associations between common dietary indices and dietary patterns defined by factor analysis and cognitive function in older community-dwelling adults. Dietary information for 1499 participants from the Rancho Bernardo Study was collected in 1988–1992 and used to calculate the alternate Mediterranean diet score, Alternate Healthy Eating Index (AHEI)-2010 score and factor scores derived from factor analysis of nutrients. Global cognitive function, executive function, verbal fluency and episodic memory were assessed at approximate four-year intervals from 1988–2016. Linear mixed models were used to examine associations between dietary patterns and cognitive trajectories. Estimates for the highest vs. lowest tertile in models adjusting for age, sex, education, energy intake, lifestyle variables and retest effect showed greater adherence to the Mediterranean score was associated with better baseline global cognitive function (β (95% CI) = 0.33 (0.11, 0.55)). The AHEI-2010 score was not significantly associated with cognitive performance. Higher loading on a plant polyunsaturated fatty acid (PUFA)/vitamin E factor was associated with better baseline global cognitive function and executive function (β = 0.22 (0.02, 0.42) and β = −7.85 (−13.20, −2.47)). A sugar/low protein factor was associated with poorer baseline cognitive function across multiple domains. Dietary patterns were not associated with cognitive decline over time. Adherence to a healthy diet with foods high in PUFA and vitamin E and a low sugar to protein ratio, as typified by a Mediterranean diet, may be beneficial for cognitive health in late life.

## 1. Introduction

As a result of an aging world population, the economic and social burden of dementia has become a global public health concern [1]. Despite significant efforts, pharmaceutical and other therapies to combat cognitive decline have thus far shown little success [2] lending more importance to identifying potentially modifiable risk factors such as diet. Although there have been numerous studies relating single nutrients to cognitive outcomes with variable results [3,4], recent approaches emphasize overall dietary patterns to address potential correlations or synergism between dietary components [5,6].

For example, adherence to the Mediterranean diet (MeDi), characterized by high intake of whole grains, legumes, vegetables and fruits with moderate consumption of alcohol and a high monounsaturated-to-saturated fat ratio, has been linked to improved cognitive function and slower cognitive decline [7,8,9]. However, these results are not consistent across study populations [10] or cognitive domains [11]. Although less studied than the MeDi, the Alternate Healthy Eating Index (AHEI), another measure of overall diet quality that reflects adherence to the Dietary Guidelines for Americans and includes specific foods and nutrients that have been shown to predict chronic disease risk [12], has also been examined with respect to cognitive performance. Results have been conflicting, with both positive [13,14] and null associations [10,15,16]. 

The use of predefined dietary indices or “a priori” approaches has several advantages because dietary index scores are easily interpreted and can be easily calculated across study populations. However, index scores may be limited when there is little variation in total index score within the population, because individuals with similar total scores may have distinct combinations of component scores reflecting very different diets. “Data-driven” approaches such as exploratory factor analysis (EFA) may offer unique insights particularly within populations where some components of dietary indices may not be frequently consumed [17]. Through EFA, a larger number of diet variables can be reduced to a set of underlying factors that explain a large proportion of the variance in the diet of the population being studied. Depending on food availability and cultural norms, there can be considerable variation in the foods typically consumed by different populations. In contrast, nutrients are to a varying degree universally consumed. Therefore, nutrient-based dietary patterns may better facilitate comparisons across divergent population groups than food-based dietary patterns [18]. Furthermore, the use of nutrient-based dietary patterns may compliment or help to explain inconsistencies in past association studies that examined single nutrient exposures only.

For health professionals it is useful to understand how currently recommended dietary patterns are related to cognitive performance, while results from data-driven approaches may help inform future dietary recommendations. Furthermore, it is important to evaluate these distinct methods within the same study cohort to minimize the effects of population-specific confounders or effect modifiers. Few, if any, previous studies have examined the relation between dietary patterns and cognitive function using both index-based and data-driven approaches simultaneously in a population with extended follow-up. This longitudinal study reports the association between dietary patterns defined by the alternate Mediterranean diet (aMed) [19], AHEI-2010 and exploratory factor analysis of nutrient intakes and cognitive performance over time among older community-dwelling adults from the Rancho Bernardo Study (RBS) of Healthy Aging. 

## 2. Materials and Methods

### 2.1. Study Participants

The Rancho Bernardo Study (RBS) of Healthy Aging is an ongoing cohort study established in 1972–1974 when 82% (*n* = 6, 339) of residents aged 30 and older, from the San Diego, CA suburb of Rancho Bernardo, were enrolled in the National Institutes of Health Lipid Research Clinic Prevalence Study of heart disease risk factors [18]. Participants were predominantly white (99.4%), middle to upper-middle class adults aged 30–79. In 1988–1992, 1727 men and women from the RBS participated in a follow-up clinic visit in which dietary information was obtained and cognitive function was initially assessed. Participants were excluded from the present study if they were less than 50 years of age at the 1988–1992 visit (*n* = 8), had missing (*n* = 169) or implausible (see below; *n* = 23) dietary information at baseline, had missing cognitive function scores at baseline (*n* = 10) or lacked information about educational attainment (*n* = 18), yielding a final sample size of 1499 participants. This study was conducted in compliance with the Declaration of Helsinki and approved by the University of California San Diego (UC San Diego) Institutional Review Board; all participants provided written informed consent prior to participation at each visit.

### 2.2. Dietary Assessment

During the 1988–1992 research clinic visit, participants completed the 153-item Willet Food Frequency Questionnaire (FFQ) [20]. Participants were asked how often, on average, during the previous year they consumed a specified common portion size for each food item. Response choices ranged from “never or less than once per month” to “six or more times per day” on a 9-point scale. The FFQ also collected information about the types of fat or oil most often used for frying, baking, and spreading.

Daily nutrient intakes were estimated using the Harvard nutrient database program by multiplying the frequency responses by the nutrient compositions of the corresponding portion sizes of each food. (HarvardSSFQ.5/93; Harvard TC Chan School of Public Health, Boston, MA, USA). Estimation of trans-fatty acid content of commonly used oils, margarines and foods was based on analyses by Litin et al. [21], Enig et al. [22] and Slover et al. [23] and from the Harvard nutrient database. Whole grain content (grams) of cold cereals and common foods was calculated using the Harvard nutrient database [24] and values from the MyPyramid Equivalents Database (MPED) [25]. FFQ results were considered implausible if 70 or more food items were left blank [26], or daily caloric intake was estimated to be lower than 600 or greater than 4200 kcal.

### 2.3. Alternate Mediterranean Diet Score

We calculated a modified Mediterranean diet score based on the scale by Trichopoulou et al. [19] as previously described [26]. We used the following components: alcohol, vegetables, legumes, fruits, nuts, whole grains, fish, red meat, and monounsaturated-to-saturated fat ratio. Individuals who reported an average alcohol intake between 5–15 g/day (approximately one 12-oz can of regular beer, 5 oz of wine, or 1.5 oz of liquor) received 1 point while those with alcohol intake outside this range received zero points. Participants with red meat intake below the sex-specific median received one point and zero points otherwise. For each of the remaining components, those consuming above the sex-specific median received one point and zero points otherwise. The sum of these component scores was used to create the aMed diet score with a range of 0–9 with a higher score representing stricter adherence to the Mediterranean diet.

### 2.4. Alternate Healthy Eating Index—2010

The AHEI score was calculated according to the AHEI-2010 score described by Chiuve et al. [27] and included the following 11 components: vegetables, fruit, whole grains, sugar-sweetened beverages and fruit juice, nuts and legumes, red/processed meat, trans-fat, long-chain (*n*-3) fats (eicosapentaenoic acid (EPA) and docosahexaenoic acid (DHA)), polyunsaturated fatty acids (PUFAs), sodium, and alcohol. Each participant received a score from 0 (worst level of intake) to 10 (best level of intake) for each component. The sum of these component scores yielded the total AHEI-2010 score with a range of 0 to 110 where a higher score represents better adherence.

### 2.5. Exploratory Factor Analysis of Dietary Nutrients

Nutrient values with a non-normal distribution were log-transformed prior to analysis. Normality of log-transformed nutrient values was assessed through visual inspection of histogram and quantile-quantile plots. Adjustment for total alcohol-free calorie intake was performed using the residual method [28]. The sample adequacy was verified by the Kaiser–Meyer–Olklin (KMO = 0.75) test and by Bartlett’s test of sphericity (*p* < 0.001) indicating that the data was appropriate for factor analysis. The following nutrients were included: vitamin B1 (thiamine), iron, vitamin B6, folate, zinc, crude fiber, beta-carotene, vitamin C, dietary cholesterol, arachidonic acid 20:4 (ω-6), vitamin B12, saturated fat, lactose, calcium, vitamin D, linoleic acid 18:2 (ω-6), alpha-linolenic acid 18:3 (ω-3), long chain *n*-3 fatty acids, vitamin E, sodium, sucrose, fructose, and protein. Nutrient intake from vitamins or other supplements was not included in this analysis. Dietary patterns were identified using EFA with the principal components analysis (PCA) extraction method. The dimensionality of the data was determined using parallel analysis where the median of eigenvalues for the correlation matrix from the nutrient data are plotted against the median of the eigenvalues calculated using a simulated uncorrelated dataset with the same number of observations and variables [29]. A factor is retained if its median eigenvalue is larger than that of the simulated data. Factors were rotated using an orthogonal transformation (varimax rotation) to improve interpretability. Nutrients with a factor loading at an absolute value ≥0.55 were regarded as contributing to that factor. Individual factor scores were calculated as a linear combination of the standardized nutrient intakes weighted by the factor loadings.

### 2.6. Cognitive Function

Cognitive function was assessed beginning at the 1988–1992 research clinic visit with continued follow-up at approximate four-year intervals. The most recent cognitive assessment occurred between 2014–2016, providing a maximum of seven assessments over a maximum 27-year period. A battery of standardized neuropsychological tests assessing global cognitive function (the Mini Mental State Exam, MMSE [30]), executive function and psychomotor processing speed (the Trail-Making Test Part B (“Trails B”) of the Halsted Reitan Battery [31], and verbal semantic fluency [32] (category fluency, assessed by number of unique animals named in one minute) was administered at each visit. A test of verbal episodic memory, the total recall score from the Buschke Selective Reminding Task [33], was administered at 5 visits; it was not given at the 1992–1997 or 2007–2009 research visits due to time constraints. Retest effects were defined as zero on the individual’s first cognitive assessment and one on all subsequent assessments [34].

### 2.7. Covariate Assessment

Lifestyle information including smoking, alcohol consumption, and exercise (≥3 times/week), was acquired through standard questionnaires at the 1988–1992 baseline visit. Participants were also asked to rate their overall health on a 5-point scale (excellent, very good, good, fair, or poor). Height and weight were measured, and BMI was calculated. Blood pressure was recorded by a trained nurse according to the Hypertension Detection and Follow-up Program protocol [35] as the mean of two readings obtained five minutes apart while the participant was in a rested, seated position. Current use of anti-hypertensive and anti-diabetic medications was obtained by questionnaire. Hypertension was defined as systolic blood pressure ≥140 mmHG or diastolic blood pressure ≥90 mmHG or use of antihypertensive medications. Fasting plasma glucose (FPG) and 2-h post-challenge plasma glucose (PCPG) were measured at a previous research clinic visit (1984–1987). Diabetes status was based on the following criteria: FPG > 126 mg/dL, PCPG > 200mg/dL [36], use of diabetes medications, or self-reported physician diagnosis.

### 2.8. Statistical Analysis

AHEI-2010 scores and factor scores were categorized into sex-specific tertiles for subsequent analyses. Individual aMed components are sex-specific [19], therefore participants were classified independently of sex into approximate aMed score tertiles. This yielded unequal group sizes due to the large number of ties in aMed scores. Descriptive statistics were calculated for baseline variables including the mean and standard deviation (SD) for continuous variables and frequency and percent for categorical variables. Differences in covariates by score tertile were assessed by chi-square analysis and ANOVAs as appropriate. We used linear mixed effects models using SAS PROC MIXED to assess the associations between dietary index or factor scores and repeated measures of the various cognitive domains. This statistical approach handles missing data and inconsistent measurement intervals within and across participants and accounts for within-subject correlation between repeated measures. Models included random intercept and time (years since baseline) effects, which allowed individual subject baseline levels and slopes to vary randomly about the mean trajectory defined by the fixed effects. Beta estimates and 95% confidence intervals were estimated using (1) a base model including time, time squared, baseline age (years), sex, education (some college; yes/no), energy intake (total calories including alcohol) and retest effects; (2) a fully adjusted model adding adjustment for potentially confounding lifestyle behaviors that may be associated with both diet and cognitive function including smoking (never/current/former), exercise (≥3 times/week; yes/no) and alcohol consumption (daily alcohol intake; yes/no). A time by dietary index/factor score interaction term was included in all models to assess the influence of dietary patterns on changes in cognitive function over time. The main effect of dietary pattern score estimates the baseline effect of the diet pattern-score (i.e., time = 0). Tests for trend across dietary pattern tertiles were carried out by assigning participants the median values of their respective dietary pattern tertile and using this as a continuous predictor in the model. Possible mediating effects of BMI, diabetes status, hypertension, and self-assessed health (fair/poor health; yes/no) were examined by assessing change in diet-score beta-estimates with and without the mediator in the model. To address bias that may result from including individuals with poor health, we repeated all analyses excluding 59 participants who rated their overall health as “fair” or “poor” compared to peers of the same age; restricting the sample to those reporting “good”, “very good”, or “excellent” health at baseline, the “healthy” subset. To account for multiple testing, we used the PROC MULTTEST procedure in SAS to calculate *q*-values, which are adjusted *p*-values controlling for the false discovery rate [37]. *q*-values < 0.05 were considered statistically significant. All analyses were carried out using SAS 9.4 (SAS Institute Inc., Cary, NC, USA).

## 3. Results

### 3.1. Participant Characteristics

Study participants had a mean age of 73.2 years (SD = 9.2) at baseline and were followed for an average of 9 (SD = 7.7) years (maximum 27 years). Women had higher AHEI-2010 scores at baseline than men (mean = 55.3, SD = 10.6 and mean = 52.7, SD = 10.4, respectively; *p* < 0.001); whereas aMed scores where similar between women and men (mean = 4.2, SD = 1.9 and mean = 4.3, SD = 1.8, respectively; *p* = 0.48). Six dietary pattern factors were identified by the EFA using the factor retention criterion from the parallel analysis. Factor loadings obtained through orthogonal rotation and the variance explained by each factor are shown in Table 1. 

We defined the factors according to the nutrients with high loadings (>0.55) as follows: (1) fortified cereals, (2) fruits and vegetables, (3) animal fat/vitamin B12, (4) dairy, (5) plant PUFA/vitamin E, and (6) sugar/low protein. In an initial factor analysis, sodium did not load highly on any factor (<0.35), and long chain *n*-3 fatty acids (EPA and DHA) loaded only moderately and to a similar extent (0.43 and 0.42) on the fruits and vegetables and animal fat/vitamin B12 factors. To improve interpretability of the factors, these nutrients were excluded from further analyses.

Baseline characteristics of participants according to aMed and AHEI-2010 dietary index score tertile and plant PUFA/vitamin E and sugar/low protein factor score tertile are shown in Table 2. Expanded tables including baseline characteristics for all dietary patterns are included in Table S1 in the supplementary material. Individuals with higher aMed or AHEI-2010 scores and those with higher fruits and vegetables factor scores tended to be more educated, exercise more and were less likely to be current smokers (*p’s* < 0.05). Higher aMed scores were also associated with increased energy intake and lower BMI (*p’s* < 0.05). Older participants were more likely to have higher scores for the fruits and vegetables and the sugar/low protein factors (*p’s* < 0.05). Those with higher sugar/low protein scores had higher daily calorie intake, were less likely to have attended college, to be married, to drink alcohol daily or to have diabetes (*p’s* < 0.05). With respect to the animal fat/vitamin B12 and the plant PUFA/vitamin E factors, individuals in the highest tertile were more likely to drink alcohol daily and had higher energy intake (*p’s* < 0.05). Increased animal fat/vitamin B12 intake was also associated with higher BMI (*p* < 0.001).

### 3.2. Dietary Pattern Scores

Beta-estimates and 95% confidence intervals (CI) for the main effects of dietary patterns from the longitudinal mixed-effects analyses for minimally and fully adjusted models for the aMed and AHEI-2010 dietary index score tertile and plant PUFA/vitamin E and sugar/low protein factor score tertile are shown in Table 3; tertile 1 is the reference level for all analyses. Expanded tables including Beta-estimates and 95% CIs for both main and interactive effects for all dietary patterns are included in Table S2 in the supplementary material. The main effects indicate baseline differences according to dietary pattern, and the dietary pattern by time interaction estimates cognitive change over time.

### 3.3. The aMed Score and Cognitive Function 

There was a significant trend for higher baseline MMSE and at higher tertiles of the aMed score in both base and full models. Verbal fluency scores were highest in individuals in the upper tertile of aMed; however, this was not significant after accounting for multiple comparisons. Baseline Trails B scores were also significantly higher for the upper tertiles of aMed, but this association did not persist after adjustment for lifestyle behaviors. aMed scores were not related to Buschke total recall performance at baseline and aMed scores were not related to the change in cognitive function test performance over time for any of the four tests (*p’s* for aMED score*time interaction ≥ 0.11).

### 3.4. The AHEI-2010 Score and Cognitive Function

The main effect of AHEI-2010 score showed a positive association with better MMSE and verbal fluency scores in minimally adjusted models only (*p* = 0.04; *q* = 0.048 and 0.01; *q* = 0.02, respectively). However, these associations were no longer significant after further adjustment for lifestyle and health-related factors. AHEI-2010 score was not significantly associated with Trails B or Buschke total recall scores. AHEI-2010 score was not significantly associated with cognitive decline over time (*p* for AHEI score*time interaction ≥ 0.13) for all tests.

### 3.5. Dietary Patterns from Exploratory Factor Analysis and Cognitive Function

**Figure 1:**
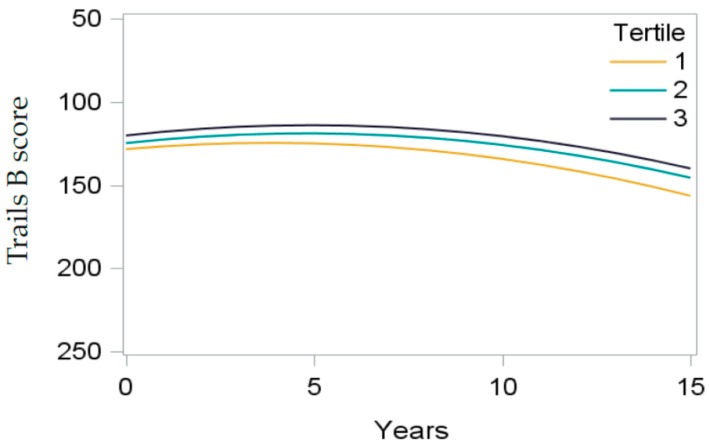
Modeled trajectories of Trails B performance over time as a function of plant polyunsatured fatty acid/vitamin E factor score tertile. Plots are based on all model coefficients using plant PUFA/vitamin E factor score tertile group-specific mean values for covariates: age, sex, education, total energy intake and practice effect. The axis for Trails B is reversed so that downward sloping lines show decreasing performance.

There were no significant main effects of the fortified cereals, fruit and vegetables, animal fat/vitamin B12, or dairy factor on cognitive test performance. After adjustment, the plant PUFA/vitamin E factor was significantly associated with MMSE and Trails B scores: participants in the highest tertile scored on average 0.22 points higher on the MMSE and 7.85 s faster on the Trails B than those in the lowest tertile (Figure 1). The sugar/low protein factor was inversely associated with baseline cognitive function across multiple domains in fully adjusted models: participants in the highest tertile had lower MMSE and verbal fluency scores than those in the lowest tertile. Increasing sugar/low protein factor scores were also associated with slower Trails B times in base models; however, this association was not significant in fully adjusted models after accounting for multiple comparisons (*p*-trend = 0.03; *q*-trend = 0.09). There were no significant differences in Buschke total recall scores according to dietary pattern factor score. None of the six factor scores were associated with significant differences in cognitive decline over time (all *p’s* ≥ 0.09).

### 3.6. Non-Participant Characteristics

Compared to study participants, non-participants (*n* = 202) were more likely to be female (69% vs. 58%; *p* = 0.003), were slightly older (74.9 ± 10.6 vs. 73.2 ± 9.2 years; *p* = 0.02), had lower body mass index (BMI) values (24.5 ± 3.6 vs. 25 ± 3.9 kg/m^2^; *p* = 0.02) and were less likely to be married (63% vs. 75%; *p* = 0.001). Participants and non-participants were similar with respect to education, exercise frequency, self-reported health and hypertension status (all *p’s* > 0.08).

### 3.7. Sensitivity Analyses

Results were similar for all index-based and factor-based diet scores when analyses were restricted to the healthy subset (data not shown). The addition of potential mediators (BMI, diabetes, hypertension, and self-assessed health (fair/poor health; yes/no) to the models showed no appreciable change in effect estimates (<10%) for index-based or factor-based diet scores.

## 4. Discussion

In this study of nearly fifteen hundred community-dwelling older adults followed over 27 years, we found small but significant diet-related differences in concurrent cognitive performance that varied by diet type and cognitive domain. Participants with greater adherence to a Mediterranean dietary pattern or higher intake of plant-based PUFA and vitamin E performed better on cognitive tasks, while those with a high sugar/low protein diet pattern demonstrated poorer cognitive test performance. In contrast, adherence to a North American healthy diet pattern (AHEI) was not robustly associated with cognitive performance. These results were independent of sex, education and lifestyle behaviors. We found no significant association between dietary patterns defined by the aMed, AHEI-2010 or nutrient factor scores and cognitive decline over time. 

Our results are in agreement with the majority of observational studies that suggest a positive association between adherence to a MeDi and cognitive performance [11,14,38,39,40]. A recent meta-analysis of 15 cohorts of older adults by Loughrey et al. [11] found that the strongest evidence was for an association between MeDi and global cognitive function; our finding that baseline MMSE score increased with aMed adherence is consistent with this. Although MeDi has previously been correlated with slower cognitive decline over time [5,7,8,9], we found no such association despite a long follow-up period. It should also be noted that we found only modest main effects, which may in part be due to differences in cultural and regional-based food preferences between US populations and the European populations from which the MeDi was derived [41]. For example the median intake of whole grains in the RBS cohort was approximately 1 serving per day, far below the recommended 8 servings/day according to the Mediterranean diet pyramid based on dietary guidelines for Greek adults [42]. Indeed, a review by Aridi et al. found that four of five studies in Mediterranean cohorts yielded significant associations between MeDi and cognitive function while only half of the 12 studies in non-Mediterranean cohorts provided significant results [40]. There are several mechanisms that may underlie the beneficial associations of MeDi with cognitive function. The MeDi has been shown to have anti-inflammatory [43] and anti-oxidant effects [44] and is associated with a decreased risk of cardiovascular disease and hypertension [45,46] both of which may contribute to cognitive decline.

Although we controlled for multiple potential confounders, it is not clear to what degree the association between the aMeD and cognitive function is driven by underlying characteristics of those individuals who decide to follow a healthy diet. However, we did not detect significant differences between those who closely followed the AHEI-2010 diet and those who did not, suggesting that the potential cognitive benefits of the aMed diet is a result of its distinct dietary components. Although the composition of the AHEI-2010 and aMed diets are similar, there are important differences; the aMed score consists of fewer components, increasing the relative contribution of common components such as nuts and alcohol. In addition, nuts and legumes are included as one component of the AHEI-2010 score, while these foods are considered separately in the aMed diet, lending them more weight. Furthermore, the sodium component, which is unique to the AHEI-2010 score, may partially mask associations as low sodium intake has previously been linked to lower cognitive function in the RBS cohort [47]. There is sparse literature examining the association between the AHEI-2010 diet and cognitive function [10,16] and no other study has had a follow-up of this length. Our results are similar to those of Haring et al. [7] and Samieri et al. [16] who found no difference in cognitive function in women according to AHEI-2010 adherence. We extend these results to a cohort including men.

Our finding that those with the highest intake of plant-based PUFAs and vitamin E have better baseline global cognitive function and executive function support previous studies linking PUFA consumption and cognitive performance [48,49,50]. In mammals, linoleic acid (LA) and alpha-linolenic acid (ALA) serve as precursors of other PUFAs of the same omega class, arachidonic acid (ARA), EPA, and DHA. PUFAs play multiple roles in brain function by influencing neurotransmission, cell survival and neuroinflammation [51] and may also mediate cognitive function through cardiovascular benefits of *n*-3 PUFAs in particular [52]. Observational studies have linked dietary intake of PUFAs to better cognitive function [53] and reduced risk of dementia and Alzheimer disease (AD) [54]. In studies by Zamroziewicz et al. serum PUFAs were positively associated with fluid intelligence, memory, gray matter volume and white matter microstructure [55,56]. Interestingly, results complimentary to ours were found in a cross-sectional study of a multi-ethnic cohort by Gu et al. [57]. Using factor analysis of dietary nutrients, investigators identified a pattern characterized by high intakes of *n*-3 and *n*-6 PUFAs and vitamin E that was positively associated with white matter integrity and cognitive function. Common dietary sources of PUFAs include seeds, nuts and vegetable oils which are also rich sources of vitamin E. The antioxidant properties of vitamin E are key in the protection of PUFAs against oxidative damage [58]. Several studies have shown that this vitamin may also have independent effects on cognitive function, though results are not entirely consistent [59]. Low plasma vitamin E has been linked to poor memory [60] and the odds of having Alzheimer’s disease (AD) [61]. However, vitamin E supplementation has not been beneficial in preventing the onset of AD or dementia in clinical trials [62,63]. It is possible that vitamin E supplementation may be more effective in combination with other important nutrients such as PUFAs. It is also important to note that this association may also reflect the synergistic effects of many nutritional components in the foods (nuts, seeds, vegetable oils) commonly consumed as part of this dietary pattern, not just the ones discussed here.

We found that higher adherence to a high sugar/low protein pattern was related to poorer performance in multiple cognitive domains after controlling for several possible lifestyle and health-related confounders. These results are consistent with previous cohort studies that revealed inverse associations between cognitive performance and total sugar intake [64] and dietary glycemic load [65]. Similarly, randomized controlled trials have demonstrated detrimental short-term effects of consuming a high glycemic load meal on memory [66,67]. A high sugar diet may over time lead to insulin resistance and diabetes [68] which is a risk factor for dementia [69]. Interestingly, the lowest rate of diabetes in this cohort was among those in the highest tertile of the high sugar/low protein pattern. It should be noted that as part of the study, participants with blood glucose values indicating diabetes were notified of their status and advised to consult their physician. Therefore, individuals with lab-identified diabetes, which was evaluated at the previous visit, would have been aware of their disease and individuals aware of their diabetes status may have changed their dietary pattern to consciously avoid high sugar foods. Previous studies report similar associations of high sugar intake with poorer cognitive function after excluding individuals with diabetes [64] or by statistical control of diabetic status [65]. Furthermore, Young et al. showed that the short-term effects of glycemic load are independent of glucose tolerance status [66]. It is also important to note that the relatively low intake of protein that characterizes this pattern may also have effects on cognitive function. Amino acids from protein-rich foods are essential for the synthesis of neurotransmitters in the brain [70]. A prospective study of elderly participants by Roberts et al. showed an increased risk of mild cognitive impairment (MCI) in those with a diet characterized by a high proportion of carbohydrates coupled with a low intake of protein [71]. Literature reviews reveal mixed results on the importance of protein intake for cognitive function [4,72] again suggesting the importance of regarding single nutrients in the context of other dietary components. We cannot exclude the possibility that the association of high sugar/low protein with poorer cognitive performance is a result of altered taste preferences that may occur within the prodromal period of cognitive impairment. Chemosensory losses of taste and smell occur with aging and are more pronounced in those with MCI or AD [73,74,75]. Indeed, individuals with AD and semantic dementia have a greater preference for sugary foods compared to non-impaired controls [76,77].

There are several limitations to this study. The demographic profile of the RBS cohort, which is predominantly white and middle class with at least some college education, may limit generalizability of these results to other populations. However, the homogeneity of the cohort increases the internal validity of our results by avoiding potential confounding effects of socioeconomic status, education and access to health care. Dietary patterns were estimated using a FFQ, and although this instrument has been previously validated [78] it is based on self-report which may have led to misclassification of dietary data. However, due to the prospective nature of this study, baseline diet recall is unlikely to have been influenced by cognitive outcomes over the follow-up period. Long chain *n*-3 fatty acids (EPA and DHA) did not load highly on one unique factor and were therefore excluded in our current analyses. Results of the FFQ suggested a relatively low intake of fish (median = 0.28 servings/day) at the time of dietary assessment. It is possible that actual fish consumption was not adequately measured by the annual FFQ which could have underestimated DHA and EPA from this dietary source. However, a prior study including a subset of participants from the RBS study population found a positive association between plasma and dietary DHA in the Rancho Bernardo population and reduced odds of AD and dementia [79]. Finally, we considered these analyses exploratory in nature and examined multiple hypotheses. After controlling for the false discovery rate associations between aMed and verbal fluency and the sugar/ low protein factor and executive function were no longer significant. However, effect sizes were small, and a larger sample size may be necessary to detect significant differences. Future studies are needed to examine reproducibility of our results in other study populations.

There are also several strengths to our study. The comprehensive data collected on this cohort allowed for control of many lifestyle and health-related confounders. This study leverages one of the longest cognitive follow-up periods (27 years) to date and includes data on multiple cognitive domains.

## 5. Conclusions

In this study of older community-dwelling adults, we found that individuals with higher adherence to the aMed diet or the PUFA/vitamin E pattern showed better performance on a test of global cognitive function, and for PUFA/vitamin E, on a test of executive function, while those with high sugar/low protein intake had poorer performance on global cognitive function and verbal fluency tests. We did not detect an association between dietary patterns based on index scores or factor analysis and cognitive decline despite an unusually long follow-up period. Given the expected increase in the prevalence of cognitive impairment world-wide, even modest effects of adherence to a healthy diet such as the MeDi may have a significant impact on global public health. Further study is needed to examine how combinations of specific dietary components such as PUFA and vitamin E influence cognitive health in other populations.

## Figures and Tables

**Table 1:** Rotated factor pattern from analysis of nutrients derived from the Willet FFQ administered to Rancho Bernardo Study participants (*n* = 1499) in 1988–1992.

	Fortified Cereals Factor	Fruit/Vegetable Factor	Animal Fat/Vit B12 Factor	Dairy Factor	Plant PUFA/Vit E Factor	Sugar/Low Protein Factor
Vit B1 (Thiamine)	**0.86**	–	–	–	–	–
Iron	**0.84**	–	–	–	–	–
Vit B6	**0.73**	0.44	–	–	–	–
Folate	**0.69**	0.50	–	–	–	–
Zinc	**0.66**	–	0.42	–	–	−0.22
Crude fiber	0.34	**0.78**	–	–	–	–
Beta-carotene	–	**0.78**	–	–	–	–
Vit C	–	**0.73**	–	–	–	0.26
Dietary Cholesterol	–	–	**0.91**	–	–	
Arachidonic acid 20:4 (ω-6)	–	–	**0.84**	–	–	−0.20
Vit B12	0.31	–	**0.66**	0.28	–	–
Saturated Fat	–	−0.46	**0.57**	–	0.42	–
Lactose	–	–	–	**0.94**	–	–
Calcium	–	–	–	**0.92**	–	–
Vit D	–	–	–	**0.81**	–	–
Linoleic acid 18:2 (ω-6)	–	–	–	–	**0.90**	–
Alpha-linolenic acid 18:3 (ω-3)	–	–	–	–	**0.87**	–
Vit E	0.49	–	–	–	**0.57**	–
Sucrose	–	–	–	–	–	**0.85**
Fructose	–	0.53	–	–	−0.21	**0.60**
Protein	–		0.53	0.25	–	**−0.65**
Variance explained (%)	16.7	13.8	13.3	12.8	10.5	8.6

Factor loadings greater than 0.55 in absolute value are bolded. Factor loadings less than 0.20 in absolute value are not shown. Vit = vitamin; PUFA = polyunsaturated fatty acid.

**Table 2:** Baseline (1988–1992) characteristics of participants according to sex-specific tertile of diet score/factor (*n* = 1499).

		Female	Mean Age	Some College	Current Smoker	Exercise ≥3x/Wk	Alcohol Daily	Fair/Poor Health ^a^	Energy Intake	BMI
	*n*	%	Years (SD)	%	%	%	%	%	kcal/day	kg/m^2^
aMed diet score										
tertile 1	525	59.4	72.7 (9.2)	65.1	12.2	62.0	37.2	5.0	1448 (473)	25.5 (4.0)
tertile 2	576	57.3	73.5 (9.6)	71.9	8.7	71.7	35.4	3.3	1667 (504)	25.2 (4.0)
tertile 3	398	58.3	73.6 (8.6)	71.6	5.3	80.1	38.9	3.5	1868 (523)	24.8 (3.4)
*p*-value		0.77	0.22	0.03	<0.001	<0.001	0.53	0.33	<0.001	0.02
AHEI-2010 score										
tertile 1	498	57.4	73.1 (9.4)	65.1	13.3	61.2	35.9	5.2	1674 (524)	25.3 (4.0)
tertile 2	507	59.0	73.6 (9.0)	71.2	9.3	73.6	37.4	3.2	1613 (519)	25.3 (4.1)
tertile 3	494	58.5	73.0 (9.1)	72.1	4.5	76.8	37.7	3.4	1645 (530)	24.9 (3.4)
*p*-value		0.88	0.51	0.03	<0.001	<0.001	0.84	0.19	0.19	0.18
Plant PUFA/vit E factor										
tertile 1	496	58.3	73.6 (8.9)	67.7	7.9	68.8	30.8	3.8	1580 (622)	25.2 (3.9)
tertile 2	500	58.4	72.9 (9.4)	69.4	9.8	73.6	39.7	4.2	1701 (503)	25.4 (4.1)
tertile 3	503	58.3	73.2 (9.2)	71.2	9.3	69.2	40.4	3.8	1650 (425)	24.9 (3.6)
*p*-value		0.99	0.50	0.5	0.54	0.17	<0.001	0.93	<0.001	0.15
Sugar/low protein factor										
tertile 1	498	58.2	70.8 (8.5)	74.3	10.8	71.9	43.6	3.0	1618 (580)	25.3 (3.8)
tertile 2	498	58.2	73.5 (9.2)	69.5	9	69.9	33.1	3.8	1617 (515)	25.4 (4.0)
tertile 3	503	58.4	75.4 (9.3)	64.6	7.2	69.9	34.3	5.0	1696 (471)	24.9 (3.7)
*p*-value		0.99	<0.001	<0.001	0.13	0.73	<0.001	0.28	0.02	0.06

All values are shown as % or mean (SD). *p*-value for differences based on ANOVA for continuous data and *χ*^2^ or Fisher’s exact test for categorical data. ^a^ Self-perceived health. aMed = alternate Mediterranean diet score; AHEI = alternate healthy eating index; Vit = vitamin; PUFA = polyunsaturated fatty acid.

**Table 3:** Parameter estimates and 95% confidence intervals from the longitudinal mixed-effects analyses of tertile of dietary pattern with cognitive function, with the lowest tertile as the reference level. *q*-values < 0.05 are noted with a * next to the *p* value.

	MMSE				Trails B				Verbal Fluency			
	Base Model ^a^		Full Model ^b^		Base Model ^a^		Full Model ^b^		Base Model ^a^		Full Model ^b^	
	Beta	95% CI	Beta	95% CI	Beta	95% CI	Beta	95% CI	Beta	95% CI	Beta	95% CI
aMed score												
tertile 2	0.22	(0.03, 0.41)	0.19	(−0.006, 0.38)	−6.10	(−7.85, −0.89)	−4.92	(−10.08, 0.25)	0.31	(−0.12, 0.75)	0.22	(−0.18, 0.62)
tertile 3	0.39	(0.17, 0.60)	0.33	(0.11, 0.55)	−5.28	(−11.11, 0.55)	−2.77	(−8.63, 3.01)	0.56	(0.06, 1.04)	0.51	(0.06, 0.97)
*p*-value	0.002 *		0.01 *		0.05		0.18		0.08		0.14	
*p*-trend	0.0003 *		0.002 *		0.06		0.32		0.02 *		0.03	
AHEI-2010 score												
tertile 2	0.22	(0.0, 0.40)	0.18	(−0.02, 0.37)	−4.33	(−9.70, 1.04)	−2.35	(−7.72, 3.02)	0.64	(0.23, 1.05)	0.46	(−0.01, 0.92)
tertile 3	0.18	(0.003, 0.37)	0.11	(−0.09, 0.31)	−3.06	(−8.45, 2.33)	−0.48	(−5.91, 4.95)	0.55	(0.13, 0.96)	0.40	(−0.05, 0.86)
*p*-value	0.04 *		0.22		0.27		0.66		0.01 *		0.10	
*p*-trend	0.13		0.17		0.80		0.85		0.01 *		0.09	
Plant PUFA/vit E												
tertile 2	0.007	(−0.20, 0.20)	−0.02	(−0.22, 0.18)	−3.90	(−9.31, 1.57)	−3.22	(−8.6, 2.6)	0.45	(−0.01, 0.90)	0.43	(−0.02, 0.88)
tertile 3	0.23	(0.02, 0.42)	0.22	(0.02, 0.42)	−8.29	(−13.70, −2.89)	−7.85	(−13.2, −2.47)	0.31	(−0.15, 0.76)	0.29	(−0.16, 0.75)
*p*-value	0.04		0.03		0.01 *		0.02 *		0.15		0.17	
*p*-trend	0.01 *		0.01 *		0.003 *		0.005 *		0.17		0.19	
Sugar/low protein												
tertile 2	−0.04	(−0.24, 0.16)	−0.03	(−0.23, 0.17)	2.13	(−3.25, 7.51)	1.34	(−4.02, 6.71)	0.04	(−0.41, 0.49)	0.05	(−0.40, 0.50)
tertile 3	−0.30	(−0.50, −0.10)	−0.29	(−0.49, −0.09)	6.94	(1.47, 12.41)	6.22	(0.76, 11.68)	−0.60	(−1.05, −0.13)	−0.57	(−1.03, −0.11)
*p*-value	0.007 *		0.008 *		0.04		0.06		0.01 *		0.01 *	
*p*-trend	0.007 *		0.01 *		0.01 *		0.03		0.03		0.04	

^a^ Base model includes time, time squared, baseline age, sex, education, energy intake, and retest effects. ^b^ Full model includes base model variables plus smoking, exercise, and alcohol consumption. * *q*-value < 0.05. aMed = alternate Mediterranean diet score; AHEI = alternate healthy eating index; Vit = vitamin; PUFA = polyunsaturated fatty acid.

## Supplementary Materials

The following are available online at http://www.mdpi.com/2072-6643/10/8/1088/s1, Table S1: Baseline (1988–1992) characteristics of participants according to sex-specific tertile of diet score/factor (*n* = 1, 499), Table S2: Parameter estimates and 95% confidence intervals from the longitudinal mixed-effects analyses of tertile of dietary pattern with cognitive function, with the lowest tertile as the reference level.
