# Supplementary material for: Dietary Patterns and Cognitive Function among Older Community-Dwelling Adults
Source: Nutrients. 2018 Aug 14;10(8):1088. doi: 10.3390/nu10081088 (PMC6116163; doi:10.3390/nu10081088)
Supplement: Supplementary file 1 [file nutrients-10-01088-s001.pdf]

Table S1. Baseline (1988–1992) characteristics of participants according to sex-specific tertile of diet score/factor (*n* = 1,499).

		Female	Mean age	Some college	Married	Current smoker	Exercise ≥3x /wk	Alcohol daily	Hypertension	Diabetes	Fair/poor health[a]	Energy intake	BMI
	n	%	Years (SD)	%	%	%	%	%	%	%	%	kcal/d	kg/m2
**aMed diet score**													
tertile 1	525	59.4	72.7 (9.2)	65.1	74.3	12.2	62.0	37.2	57.9	12.0	5.0	1448 (473)	25.5 (4.0)
tertile 2	576	57.3	73.5 (9.6)	71.9	74.3	8.7	71.7	35.4	63.4	14.5	3.3	1667 (504)	25.2 (4.0)
tertile 3	398	58.3	73.6 (8.6)	71.6	75.1	5.3	80.1	38.9	60.0	11.5	3.5	1868 (523)	24.8 (3.4)
p-value		0.77	0.22	0.03	0.95	<0.001	<0.001	0.53	0.17	0.31	0.33	<0.001	0.02
**AHEI-2010 score**													
tertile 1	498	57.4	73.1 (9.4)	65.1	73.3	13.3	61.2	35.9	58.9	11.3	5.2	1674 (524)	25.3 (4.0)
tertile 2	507	59.0	73.6 (9.0)	71.2	75.0	9.3	73.6	37.4	63.9	13.4	3.2	1613 (519)	25.3 (4.1)
tertile 3	494	58.5	73.0 (9.1)	72.1	75.3	4.5	76.8	37.7	58.9	13.7	3.4	1645 (530)	24.9 (3.4)
p-value		0.88	0.51	0.03	0.74	<0.001	<0.001	0.84	0.18	0.47	0.19	0.19	0.18
**Fortified cereals factor**													
tertile 1	495	58.8	73.3 (9.4)	68.7	74.7	9.9	68.9	38.5	61.9	11.8	5.1	1609 (647)	25.0 (3.6)
tertile 2	503	58.1	72.7 (9.3)	67.4	76.1	8.7	73.1	35.6	58.9	12.1	4.4	1674 (445)	25.2 (4.0)
tertile 3	501	58.1	73.7 (8.9)	72.3	72.7	8.4	69.5	36.9	60.8	14.3	2.4	1649 (458)	25.3 (3.9)
p-value		0.97	0.25	0.22	0.44	0.68	0.29	0.64	0.62	0.43	0.08	0.14	0.75
**Fruits and vegetables factor**													
tertile 1	498	58.4	72.5 (9.2)	64.9	74.9	14.3	62.2	39.6	57.2	13.1	4.4	1609 (547)	25.2 (4.0)
tertile 2	502	58.4	73.1 (9.1)	72.3	75.3	7.8	71.7	37.1	62.8	12.3	4.0	1681 (536)	25.3 (3.9)
tertile 3	499	58.1	74.1 (9.1)	71.1	73.3	5.0	77.6	34.3	61.6	12.6	3.4	1641 (487)	25.2 (3.7)
p-value		0.99	0.02	0.02	0.76	<0.001	<0.001	0.21	0.16	0.92	0.71	0.09	0.90
**Animal fat/ vit B12 factor**													
tertile 1	496	58.7	74.0 (9.0)	65.9	73.2	8.7	69.2	32.3	59.4	13.1	4.8	1592 (600)	24.6 (3.5)
tertile 2	500	57.8	72.9 (9.2)	72.2	74.6	10.2	71.5	37.6	59.6	12.4	3.0	1681 (524)	25.2 (3.8)
tertile 3	503	58.4	72.8 (9.3)	70.2	75.7	8.2	70.9	41.0	62.5	12.5	4.0	1658 (434)	25.8 (4.1)
p-value		0.96	0.07	0.09	0.65	0.5	0.7	0.02	0.52	0.93	0.33	0.02	<0.001
**Dairy factor**													
tertile 1	500	58.2	72.8 (9.0)	67.6	77.2	11.0	68.9	40.4	63.2	10.8	3.4	1561 (569)	25.3 (3.7)
tertile 2	502	58.4	73.9 (9.6)	66.9	73.5	7.2	71.2	35.5	57.8	13	4.2	1726 (525)	25.3 (3.9)
tertile 3	497	58.4	73.0 (8.9)	73.8	72.8	8.9	71.5	35.1	60.5	14.4	4.2	1644 (462)	25.0 (4.0)
p-value		0.99	0.15	0.03	0.23	0.11	0.63	0.15	0.21	0.22	0.75	<0.001	0.46
**Plant PUFA/ vit E factor**													
tertile 1	496	58.3	73.6 (8.9)	67.7	72.0	7.9	68.8	30.8	63.1	13.0	3.8	1580 (622)	25.2 (3.9)
tertile 2	500	58.4	72.9 (9.4)	69.4	76.6	9.8	73.6	39.7	60.0	10.9	4.2	1701 (503)	25.4 (4.1)
tertile 3	503	58.3	73.2 (9.2)	71.2	75.0	9.3	69.2	40.4	58.6	15.6	3.8	1650 (425)	24.9 (3.6)
p-value		0.99	0.50	0.5	0.24	0.54	0.17	<0.001	0.33	0.07	0.93	<0.001	0.15
**Sugar/ low protein factor**													
tertile 1	498	58.2	70.8 (8.5)	74.3	78.1	10.8	71.9	43.6	58.6	15.4	3.0	1618 (580)	25.3 (3.8)
tertile 2	498	58.2	73.5 (9.2)	69.5	74.1	9	69.9	33.1	61.4	12.0	3.8	1617 (515)	25.4 (4.0)
tertile 3	503	58.4	75.4 (9.3)	64.6	71.4	7.2	69.9	34.3	61.6	10.6	5.0	1696 (471)	24.9 (3.7)
p-value		0.99	<0.001	<0.001	0.048	0.13	0.73	<0.001	0.54	0.07	0.28	0.02	0.06

All values are shown as % or mean (SD).
P-value for differences based on ANOVA for continuous data and $\chi 2$ or Fisher's exact test for categorical data.
[a]Self-perceived health
aMed=alternate Mediterranean diet score, AHEI=alternate healthy eating index; Vit=vitamin;
PUFA=polyunsaturated fatty acid

Table S2. Parameter estimates and 95% confidence intervals from the longitudinal mixed-effects analyses of tertile of dietary pattern with cognitive function, with the lowest tertile as the reference level.

	**MMSE**				**Trails B**				**Verbal Fluency**				**Buschke Total Recall**			
	**Base Model[a]**		**Full Model[b]**		**Base Model[a]**		**Full Model[b]**		**Base Model[a]**		**Full Model[b]**		**Base Model[a]**		**Full Model[b]**	
	Beta	95%CI	Beta	95%CI	Beta	95%CI	Beta	95%CI	Beta	95%CI	Beta	95%CI	Beta	95%CI	Beta	95%CI
**aMed score**																
tertile 2	0.22	(0.03, 0.41)	0.19	(-0.006,0.38)	-6.10	(-7.85,-0.89)	-4.92	(-10.08,0.25)	0.31	(-0.12,0.75)	0.22	(-0.18,0.62)	0.98	(0.10,1.90)	0.87	(-0.01,1.75)
tertile 3	0.39	(0.17,0.60)	0.33	(0.11,0.55)	-5.28	(-11.11,0.55)	-2.77	(-8.63,3.01)	0.56	(0.06,1.04)	0.51	(0.06,0.97)	0.54	(-0.45,1.54)	0.26	(-0.75,1.26)
P-value	0.002		0.01		0.05		0.18		0.08		0.14		0.10		0.13	
P-trend	0.0003		0.002		0.06		0.32		0.02		0.03		0.28		0.62	
**aMed *time**																
tertile 2	-0.004	(-0.03,0.03)	-0.004	(-0.03,0.03)	0.49	(-0.37,1.35)	0.50	(-0.35,1.34)	-0.04	(-0.11,0.03)	-0.03	(-0.08,0.02)	0.002	(-0.15,0.15)	-0.002	(-0.15,0.15)
tertile 3	-0.03	(-0.06,0.01)	-0.03	(-0.06, 0.01)	0.13	(-0.86,1.11)	0.06	(-0.90,1.03)	-0.003	(-0.08,0.08)	-0.01	(-0.07,0.05)	0.06	(-0.11,0.24)	0.07	(-0.11, 0.24)
P-value	0.18		0.20		0.51		0.46		0.39		0.55		0.73		0.69	
P-trend	0.11		0.12		0.71		0.79		0.82		0.62		0.48		0.46	
**AHEI-2010 score**																
tertile 2	0.22	(0.0,0.40)	0.18	(-0.02,0.37)	-4.33	(-9.70,1.04)	-2.35	(-7.72,3.02)	0.64	(0.23,1.05)	0.46	(-0.01,0.92)	0.60	(-0.30,1.52)	0.45	(-0.47,1.36)
tertile 3	0.18	(0.003,0.37)	0.11	(-0.09,0.31)	-3.06	(-8.45,2.33)	-0.48	(-5.91,4.95)	0.55	(0.13,0.96)	0.40	(-0.05,0.86)	0.21	(-0.71,1.20)	-0.001	(-0.92,0.93)
P-value	0.04		0.22		0.27		0.66		0.01		0.10		0.41		0.53	
P-trend	0.13		0.17		0.80		0.85		0.01		0.09		0.57		0.98	
**AHEI-2010 score*time**																
tertile 2	-0.01	(-0.03,0.02)	-0.02	(-0.04,0.02)	-0.12	(-1.03,0.81)	-0.19	(-1.10,0.71)	-0.03	(-0.09,0.03)	0.002	(-0.07,0.08)	0.07	(-0.08,0.24)	0.08	(-0.08,0.24)
tertile 3	-0.02	(-0.05,0.01)	-0.01	(-0.05,0.01)	0.27	(-0.65,1.18)	0.09	(-0.81,0.99)	0.02	(-0.04,0.08)	0.05	(-0.03,0.12)	0.06	(-0.109,0.22)	0.07	(-0.09,0.23)
P-value	0.28		0.22		0.70		0.81		0.41		0.36		0.65		0.57	
P-trend	0.13		0.15		0.83		0.81		0.38		0.18		0.62		0.49	
**Fortified cereals**																
tertile 2	0.03	(-0.17,0.22)	0.03	(-0.18,0.22)	-0.34	(-5.72,5.03)	0.17	(-5.30,5.34)	0.16	(-0.29,0.61)	0.16	(-0.30,0.61)	0.75	(-0.15,1.66)	0.72	(-0.19,1.63)
tertile 3	0.15	(-0.05,0.35)	0.13	(-0.07,0.33)	-0.35	(-5.80,5.07)	-0.16	(-5.90,5.54)	-0.11	(-0.56,0.34)	-0.13	(-0.58,0.33)	-0.16	(-1.07,0.76)	-0.17	(-1.10,0.73)
P-value	0.30		0.36		0.99		0.99		0.50		0.51		0.11		0.12	
P-trend	0.08		0.10		0.89		0.89		0.66		0.62		0.61		0.73	
**Fortified cereals*time**																
tertile 2	-0.02	(-0.05,0.01)	-0.02	(-0.05,0.01)	0.85	(-0.07,1.77)	0.85	(-0.05,1.75)	-0.03	(-0.10,0.05)	-0.03	(-0.10,0.05)	-0.06	(-0.23,0.10)	-0.06	(-0.22,0.10)
tertile 3	-0.01	(-0.04,0.03)	-0.01	(-0.04,0.02)	0.31	(-0.60,1.22)	0.35	(-0.54,1.24)	0.006	(-0.07,0.08)	0.006	(-0.07,0.08)	0.14	(-0.03,0.3)	0.13	(-0.03,0.29)
P-value	0.53		0.55		0.18		0.18		0.68		0.68		0.07		0.09	
P-trend	0.72		0.71		0.63		0.21		0.90		0.91		0.07		0.09	
**Fruits and vegetables**																
tertile 2	0.15	(-0.05,0.34)	0.12	(-0.08,0.32)	-5.92	(-12.30,-0.46)	-6.08	(-13.0,-0.43)	-0.11	(-0.56,0.34)	-0.14	(-0.59,0.31)	-0.65	(-1.56,0.26)	-0.79	(-1.71,0.12)
tertile 3	0.15	(-0.05,0.35)	0.09	(-0.11,0.30)	1.44	(-4.98,7.87)	1.04	(-5.37,6.83)	0.29	(-0.16,0.75)	0.21	(-0.25,0.67)	-0.75	(-1.66,0.17)	-1.00	(-2.01,0.01)
P-value	0.25		0.48		0.06		0.06		0.20		0.30		0.22		0.10	
P-trend	0.13		0.33		0.62		0.82		0.30		0.47		0.14		0.07	
**Fruits and vegetables*time**																
tertile 2	-0.01	(-0.03,0.03)	-0.002	(-0.03,0.03)	0.17	(-0.52,0.87)	0.20	(-0.49,0.68)	-0.03	(-0.10,0.04)	-0.04	(-0.11,0.04)	-0.01	(-0.17,0.15)	0.004	(-0.15,0.16)
tertile 3	-0.03	(-0.06,0.01)	-0.02	(-0.05,0.01)	-0.06	(-0.78,0.67)	0.04	(-0.69,0.77)	-0.02	(-0.09,0.06)	-0.01	(-0.09,0.06)	-0.05	(-0.21,0.12)	-0.03	(-0.19,0.13)
P-value	0.24		0.32		0.80		0.82		0.68		0.69		0.81		0.87	
P-trend	0.11		0.16		0.50		0.87		0.97		0.94		0.39		0.87	

Table S2. Cont.

**Animal fat/ vit B12**																
tertile 2	0.05	(-0.15,0.26)	0.05	(-0.16,0.25)	-5.44	(-10.90,0.16)	-4.86	(-10.26,0.53)	0.26	(-0.20,0.71)	0.24	(-0.21,0.70)	0.20	(-0.72,1.11)	0.14	(-0.78,1.05)
tertile 3	0.09	(-0.11,0.29)	0.12	(-0.08,0.32)	-4.41	(-9.84,0.99)	-3.94	(-9.32,1.43)	0.15	(-0.30,0.61)	0.18	(-0.28,0.63)	0.53	(-0.38,1.45)	0.46	(-0.45,1.37)
P-value	0.67		0.50		0.12		0.17		0.54		0.56		0.51		0.59	
P-trend	0.21		0.26		0.11		0.15		0.35		0.30		0.28		0.35	
**Animal fat/ vit B12*time**																
tertile 2	-0.01	(-0.04,0.02)	-0.01	(-0.04,0.02)	0.64	(-0.28,1.57)	0.66	(-0.25,1.57)	-0.07	(-0.14,0.01)	-0.06	(-0.14,0.01)	-0.11	(-0.27,0.05)	-0.11	(-0.27,0.05)
tertile 3	0.001	(-0.03,0.03)	0.001	(-0.03,0.03)	0.06	(-0.86,0.99)	0.04	(-0.87,0.95)	-0.03	(-0.10,0.05)	-0.03	(-0.10,0.05)	-0.04	(-0.20,0.13)	-0.03	(-0.19,0.14)
P-value	0.60		0.72		0.30		0.26		0.54		0.23		0.39		0.37	
P-trend	0.99		0.95		0.89		0.95		0.33		0.35		0.88		0.98	
**Dairy**																
tertile 2	0.08	(-0.12,0.28)	0.07	(-0.13,0.26)	-1.36	(-6.78,4.05)	-1.10	(-6.49,4.29)	0.36	(-0.09,0.82)	0.37	(-0.09,0.83)	-0.41	(-1.32,0.51)	-0.42	(-1.33,0.50)
tertile 3	-0.02	(-0.22,0.17)	-0.34	(-0.23,0.17)	1.59	(-3.79,6.97)	1.89	(-3.46,7.25)	-0.02	(-0.48,0.43)	-0.06	(-0.51,0.39)	-0.007	(-0.92,0.91)	0.001	-0.91,0.91)
P-value	0.59		0.62		0.56		0.54		0.18		0.13		0.61		0.60	
P-trend	0.71		0.67		0.63		0.53		0.90		0.77		0.96		0.90	
**Dairy*time**																
tertile 2	0.001	(-0.03,0.03)	0.002	(-0.03,0.03)	0.29	(-0.64,1.22)	0.25	(-0.65,1.16)	-0.03	(-0.11,0.04)	-0.03	(-0.10,0.05)	0.03	(-0.14,0.19)	0.03	(-0.13,0.19)
tertile 3	0.01	(-0.02,0.04)	0.008	(-0.02,0.04)	0.02	(-0.90,0.94)	-0.04	(-0.93,0.86)	-0.007	(-0.08,0.07)	-0.005	(-0.08,0.07)	0.04	(-0.13,0.20)	0.03	(-0.13,0.19)
P-value	0.83		0.84		0.80		0.54		0.68		0.69		0.91		0.92	
P-trend	0.53		0.52		0.97		0.89		0.86		0.91		0.72		0.70	
**Plant PUFA/ vit E**																
tertile 2	0.007	(-0.20,0.20)	-0.02	(-0.22,0.18)	-3.90	(-9.31,1.57)	-3.22	(-8.6,2.6)	0.45	(-0.01,0.90)	0.43	(-0.02,0.88)	0.55	(-0.37,1.47)	0.44	(-0.48,1.35)
tertile 3	0.23	(0.02,0.42)	0.22	(0.02,0.42)	-8.29	(-13.70,-2.89)	-7.85	(-13.2,-2.47)	0.31	(-0.15,0.76)	0.29	(-0.16,0.75)	0.67	(-0.25,1.59)	0.63	(-0.29,1.54)
P-value	0.04		0.03		0.01		0.02		0.15		0.17		0.31		0.39	
P-trend	0.01		0.01		0.003		0.005		0.17		0.19		0.15		0.19	
**Plant PUFA/ vit E*time**																
tertile 2	0.04	(0.005,0.07)	0.04	(0.005,0.07)	-0.48	(-1.40,0.45)	-0.47	(-1.39,0.43)	0.07	(-0.005,0.14)	0.07	(-0.003,0.14)	0.17	(0.01,0.34)	0.17	(0.01,0.33)
tertile 3	0.03	(-0.01,0.06)	0.02	(-0.01,0.05)	-0.59	(-1.49,0.32)	-0.50	(-1.39,0.39)	0.06	(-0.01,0.14)	0.07	(-0.01,0.14)	0.14	(-0.02,0.30)	0.13	(-0.03,0.29)
P-value	0.07		0.07		0.42		0.48		0.12		0.11		0.10		0.10	
P-trend	0.21		0.22		0.20		0.27		0.09		0.08		0.13		0.14	
**Sugar/ low protein**																
tertile 2	-0.04	(-0.24,0.16)	-0.03	(-0.23,0.17)	2.13	(-3.25,7.51)	1.34	(-4.02,6.71)	0.04	(-0.41,0.49)	0.05	(-0.40,0.50)	-0.07	(-0.99,0.84)	-0.04	(-0.95,0.88)
tertile 3	-0.30	(-0.50,-0.10)	-0.29	(-0.49,-0.09)	6.94	(1.47,12.41)	6.22	(0.76,11.68)	-0.60	(-1.05,-0.13)	-0.57	(-1.03,-0.11)	-0.88	(-1.81,0.05)	-0.89	(-1.81,0.04)
P-value	0.007		0.008		0.04		0.06		0.01		0.01		0.11		0.10	
P-trend	0.007		0.01		0.01		0.03		0.03		0.04		0.06		0.06	
**Sugar/ low protein*time**																
tertile 2	-0.01	(-0.05,0.02)	-0.02	(-0.05,0.01)	0.03	(-0.85,0.92)	0.05	(-0.83,0.92)	0.004	(-0.07,0.08)	-0.001	(-0.07,0.07)	0.02	(-0.13,0.18)	0.02	(-0.13,0.18)
tertile 3	0.006	(-0.03,0.04)	0.007	(-0.02,0.04)	0.10	(-0.82,1.02)	0.06	(-0.84,0.97)	0.008	(-0.07,0.08)	0.004	(-0.07,0.08)	-0.007	(-0.17,0.16)	0.001	(-0.16,0.16)
P-value	0.42		0.37		0.98		0.98		0.98		0.99		0.93		0.95	
P-trend	0.81		0.78		0.84		0.92		0.96		0.96		0.78		0.72	

[a]Base model includes time, time squared, baseline age, sex, education, energy intake, and retest effects.

[b]Full model includes base model variables plus smoking, exercise, and alcohol consumption.

aMed= alternate Mediterranean diet score, AHEI= alternate healthy eating index; Vit = vitamin; PUFA = polyunsaturated fatty acid.
